# PFKFB4 is overexpressed in clear-cell renal cell carcinoma promoting pentose phosphate pathway that mediates Sunitinib resistance

**DOI:** 10.1186/s13046-021-02103-5

**Published:** 2021-09-30

**Authors:** Chenchen Feng, Yuqing Li, Kunping Li, Yinfeng Lyu, Wenhui Zhu, Haowen Jiang, Hui Wen

**Affiliations:** 1 Department of Urology, Huashan Hospital, Fudan University, 12 Middle Urumqi Rd, 200040 Shanghai, PR China; 2 Institute of Urology, Fudan University, 200040 Shanghai, PR China

**Keywords:** Clear-cell renal cell carcinoma, PFKFB4, Pentose phosphate pathway, Sunitinib, Resistance

## Abstract

**Background:**

Kinases play critical role in clear-cell renal cell carcinoma (ccRCC). We aim to exploit novel kinase that is both protumorigenic and drugable in ccRCC.

**Methods:**

Reproduction of public datasets with validation using microarray was performed to identify candidate gene. Functionality was studied using multi-omics with validation in vitro and in vivo.

**Results:**

6-Phosphofructo-2-Kinase/Fructose-2,6-Biphosphatase 4 (PFKFB4) was differentially expressed showing significantly higher expression in tumor than in normal kidney. PFKFB4 overexpression was associated with advanced tumor grade, stage and worsened prognosis. PFKFB4-knockdown significantly impaired fitness in cell proliferation, migration and wound healing. Despite being recurrently deleted on 3p, PFKFN4 mRNA remained actively transcribed by HIF1α. Metabolomics showed overexpressed PFKFB4 showed enriched metabolites in pentose phosphate pathway (PPP). Phosphoproteomics and immunoprecipitation showed PFKFB4 also phosphorylated NCOA3 which interacted with FBP1 to counteract overactive PPP flux, forming a regulatory loop. PFKFB4-knockdown overcame resistance to Sunitinib in vitro and in vivo both in xenograft and tail-vein injection murine models.

**Conclusion:**

We concluded PFKFB4 was associated with PPP activity and the fine-tuning of which was mediated by its phosphorylation of NCOA3. Targeting PFKFB4 held promise to combat resistance to Sunitinib.

**Supplementary Information:**

The online version contains supplementary material available at 10.1186/s13046-021-02103-5.

## Background

Renal cell carcinoma (RCC) originates from renal tubular epithelial cells, of which age-standardized incidence is on average 4.4/100,000 around the world and causes more than 140,000 deaths annually [1]. Clear-cell renal cell carcinoma (ccRCC), the most common pathological subtype, accounts for more than 90 % of newly diagnosed RCC cases [2]. Insightful understanding of molecular biology of ccRCC therefore holds promise for novel treatment development.

Reprogramming of glucose metabolism is a hallmark of cancer by which cancer cells hijack energy to meet needs for rapid growth [3, 4]. Rewiring of glucose metabolism also plays pivotal role in ccRCC. Over a decade ago, differential level of enzymes of glycolysis and pyruvate metabolism in urine have already been reported in ccRCC patients [5]. Metabolites that participate in glycolysis, such as glucose 6-phosphate (G6P), and fructose 6-phosphate (F6P) showed over 2-fold increase, underscoring metabolic alteration in ccRCC. Later, isotope assays validated enriched products from glycolysis and decreased metabolites of Krebs cycle, supporting prevalent “Warburg effect” in ccRCC. In the era of next-generation sequencing, metabolic reprogramming has been closely associated with truncal genetic events like loss of 3p genes, i.e. VHL and activation of hypoxia-inducible factor (HIF)-1α/2α, further corroborating the pivotal role of metabolic rewiring [6–8].

Apart from glycolysis, activation of several metabolic shunts has also been reported to play a role in ccRCC. Expressions of pentose phosphate pathway (PPP) genes has been reported to correlated with survival outcome in ccRCC [9]. Lipogenesis and altered glutamine metabolism have also been reported to be a major source for energy supply and efficient approach to clear free radicals in ccRCC under hypoxia [10–12]. All those findings indicate that insightful understanding of glucose metabolism is of importance in ccRCC.

Phosphofructokinase 2 (PFK2), which presents four active forms: 6-phosphofructo-2-kinase/fructose-2,6-bisphosphatase (PFKFB) 1–4, is a rate-limiting enzyme that catalyzes the Fructose 6-phosphate (F6P) to fructose 1,6-bisphosphate (F-1,6-2P). Also as a kinase, it retains the activity of phosphatase as well [13]. All four types of isoenzymes are activated upon hypoxia. PFKFB4 is reported to be a robust stimulator to nuclear receptor coactivator 3 (NCOA3) which drives glucose flux towards the PPP and up-regulates the activity of estrogen receptor to further promote aggressiveness of breast cancer [14]. In small-cell lung cancer, PFKFB4 has been found to be a downstream target and interacting protein of endothelial tyrosine kinase to promote the chemoresistance to ibrutinib by regulating autophagy [14]. Altogether, PFKFB4 has been reported to be pro-tumorigenic is several solid tumors and is considered to enhance glycolytic flux [15, 16]. However, its role in ccRCC has not been reported.

In the current study, we have carried out series of assays in silico, in tissue, in vitro and in vivo to comprehensively evaluate role of PFKFB4 in ccRCC. Our findings hold promise to better understand biology of ccRCC and to development of novel treatment.

## Materials and methods

### Bioinformatics and statistical analysis

We utilized public datasets including TCGA (https://portal.gdc.cancer.gov/), ICGC (https://icgc.org/) and GEO (https://www.ncbi.nlm.nih.gov/geo/) to extract expression profiling and clinical information of renal cell carcinoma patients. 1633 over-expressed genes of TCGA-KIRC were acquired from GEPIA database [17] (Gene Expression Profiling Interactive Analysis, http://gepia.cancer-pku.cn/ ); LIMMA was used to differential analysis, |Log_2_FC| cutoff and q-value cutoff were 1 and 0.01, respectively. All the informatic analysis were performed on R studio software (version 4.0.2). The mRNA data were normalized to TPM format, and were compared between tumor and adjacent or normal specimen using student t test and ggplot2 package. Survival analysis and multivariate cox regression model were conducted by survival and timeROC packages. 2195 kinase genes were downloaded from the Human Protein Atlas ( https://www.proteinatlas.org/ ). The gene expression correlation analysis and genetic alteration analysis were performed via the cBioPortal database [18] (https://www.cbioportal.org/). GEPIA database and Cancer Cell Line Encyclopedia database [19] (CCLE, https://portals.broadinstitute.org/ccle ) were employed to gene expression in pan-cancer. Toolkit for Cistrome Data Browser [20] (http://dbtoolkit.cistrome.org/) was utilized to calculate and further visualize the regulatory potential(RP) scores of differential expression genes based on the ChIP-seq data.

### Selection of candidate genes

The over-expressed genes from TCGA-KIRC and our own sequencing data were intersected, from which the overexpressed-kinase gene was then picked out by intersected with kinase genes. Univariate COX regression of overall survival (OS) and progress-free survival (PFS) was conducted to screen prognostic related genes. Then area under curve (AUC) values of OS-related unfavorable genes were calculated and the scatter plot was used to visualized it. The forest plot of PFS-related unfavorable genes was drawn. 5 candidate genes could be picked out from above two plots. Pearson correlation was used to analyze the correlation of gene expression.

### mRNA microarray

Nineteen Paired tumor and normal samples were collected from patients with clear-cell renal cell carcinoma during November 2018 to February 2019 in Huashan Hospital affiliated to Fudan University. All specimens were preserved at -196℃ with liquid nitrogen. The total RNA of the samples was extracted by Trizol, and was inspected by Nanodrop 2000 and Agilent BioAnalyzer 2100 for quality inspection. To satisfy the criterion of quality, RNA was processed with Pico Reagent Kit. The mRNA samples were then converted into complementary DNA (cDNA), the fragments of which were labeled with DNA marker and attached to biotin. The biotin-labeled cRNA was hybridized on Affymetrix microarray chip to detect approximately 50,000 probes. The results were scanned using GeneChip Scanner 3000. Normalized data were presented in Supplementary Table 1.

### Metabolomics

A standard protocol for metabolite analyzing was followed. 786O cells with PFKFB4-knockdown or control were cultured for 48 h and were washed twice with chilled phosphate buffer saline (PBS) and once with 0.9 % NaCl solution. Cells were then quenched with liquid nitrogen and scraped with addition of Methanol/acetonitrile/water at 2:2:1 (v/v). Six samples for biological duplicates and 4 samples for quality control (QC) was used. Samples were then centrifuged at 14,000 g for 15 min at 4 °C and vac evaporation was performed. Samples were then resuspended with acetonitrile/water at 2:1 (v/v) and the supernatant and precipitates were processed to the Agilent 1290 Infinity LC ultra-high-performance liquid chromatography (UHPLC) system for further analysis. Randomized sampling was performed with one QC sample separating every 5 testing samples. The HILIC column and HSS T3 column were used for LC separation using gradient elution. Metabolites were then detected with electrospray ionization (ESI), examining metabolites in both positive and negative ion modes (AB SCIEX). The XCMS software was used to analyze the iron current of each metabolite and the Metaboanalyst was used to perform multidimensional statistical analyses including unsupervised PCA and PLS-DA. The R package was used to study volcano distribution. The Kyoto Encyclopedia of Genes and Genome (KEGG) pathway database was exploited to perform the Metabolite Set Enrichment Analysis (MSEA). Normalized data were presented in Supplementary Table 2.

### Phosphoproteomics

The Tandem Mass Tag (TMT) technique was used to study phosphoproteomics in PFKFB4-overexpressed (OE) and control 786O cells. After protein lysate was prepared, samples were subject to SDS-PAGE electrophoresis and Filter aided proteome preparation (FASP) in which C18 cartridge was used for desalting. 100 µg of peptide was then marked using TMT kit (Thermo) as per manufacturer’s protocol. Peptides were then processed for enrichment of phosphopeptides and were subject to Easy nLC chromatography with 1 h gradient. After separation by chromatography, samples were analyzed by Q Exactive plus mass spectrometer. Normalized data were presented in Supplementary Table 3.

### Cell lines and RNA interference

786O, A498, Caki1 and RCC4 ccRCC cancer cells were obtained from CellScource China. Cells were cultured in RPMI-1640 medium supplemented with 10 % of FBS. The GPP Web Portal (https://portals.broadinstitute.org/gpp/public/) was used for shRNA construction (Supplementary Table 4). Scrambled shRNAs were used as negative control (NC). cDNA clone for PFKFB4, HIF1A, NCOA3 and FBP1 were obtained from Origene. Overexpression was realized by adenoviral or lentiviral delivery using polybrene system. Quantitative PCR was performed to examine the shRNA effect and constitutive PFKFB4 expression level in different ccRCC cell lines. Generation of Sunitinib –sensitive and –resistant cell lines was according established protocols [21]. Briefly, sunitinib-resistant 786O cells were generated via prolonged exposure to 10 µM sunitinib, and subcultured every 3–4 d for > 20 passages. Short exposure in the current study was defined as Sunitinib treatment of 96 h at indicated dose of IC50. Primers were constructed using the PrimerBank (https://pga.mgh.harvard.edu/primerbank/) and were listed in Supplementary Table 4. Treatment of Sunitinib and 5MPN were respectively indicated in figure legends of different assays.

### Western blotting

Western blot was carried out according to the standard protocol and protein lysates were acquired from cultured cells treated differently. 10 % SDS-PAGE was used to isolate proteins and then transferred to a nitrocellulose membrane. After being sealed with skimmed milk at room temperature for nearly 1 h, the membranes were incubated at 4 °C overnight. Antibodies used were listed in Supplementary Table 4. Then the ECL system was used to detect the immune response bands according to the manufacturing instructions. Image J 1.47 V software (http://imagej.nih.gov/ij) was utilized for densitometry measurements.

### Immunohistochemistry (IHC)

A total of 324 formalin-fixed paraffin-embedded (FFPE) ccRCC Sec. (5 μm) archived in our tissue bank were stained with hematoxylin-eosin to observe tumor morphology [22]. The xylene-deparaffinized and rehydrated sections were conducted heat-mediated antigen retrieval in citric acid buffer (pH 6.0) with microwave for 30 min to IHC staining. Sections were inactivated by endogenous peroxidase for 10 min (3 % H_2_O_2_) and blocked by non-specific binding, then incubated overnight with diluted primary antibodies at 4 °C. Next, sections were continuously incubated at room temperature with biotinylated secondary antibodies and streptavidin horseradish peroxidase. The standard DAB staining and hematoxylin counterstaining were used to observe the antigen binding. Light microscope was used to take images. Antibodies used were listed in Supplementary Table 4.

### Co-Immunoprecipitation (Co-IP)

786O cells with lentiviral PFKFB4-OE or control were prepared and examine for protein level by western blotting of Flag (Sigma, F1804, mouse, at 1:1000). Cells were rinsed with PBS twice and lysed pre-chilled. Cells were fragmented by ultrasound and protein concentration was determined by BCA method. Load EP tube with Flag beads and add protein lystes to a total of 1200 µl/tube. After incubation overnight at 4 °C, samples were centrifuged. Candidate genes were pre-selected by shotgun proteomics using high performance liquid chromatography combined with mass spectrometry (MS) using Q Executive for differentially translated proteins of interest. We designated unique peptide of 1 or above as credible proteins. Genes of interest were subject to western blotting in the IP assay and western blotting was performed. Antibodies used were listed in Supplementary Table 4.

### RNA isolation and quantitative PCR (qPCR)

We used TRIzol reagent (Invitrogen) to extract the total RNA of the cells according to the instructions of the manufacturer. PrimeScript™ RT Master Mix (TakaRa) was employed to perform reverse transcription reactions of RNA samples. For determining the expression levels of cDNA, SYBR® Premix ExTaq™ II (TaKaRa) was used to conduct quantitative real-time polymerase chain reaction (qRT-PCR) analyses according to manufacturer’s protocols. The internal control in this experiment was GAPDH. Ct method was used to calculate the relative abundance of mRNA after normalization. The primer pairs for qPCR analysis were listed the Supplementary Table 4.

### Chromatin immunoprecipitation (ChIP)-PCR analysis

Cells with a concentration of 2 million /mL were treated with 1 % formaldehyde for 10 min at room temperature. After being washed twice with ice PBS containing protease inhibitors, the cells were centrifuged into pellets and resuspend in SDS lysis buffer for incubating at 4 °C for 15 min, followed by sonicated 12 times (30 s each). After centrifugation, the supernatant was added with ChIP dilution buffer and protein G beads. The DNA fragments were pulled down by the antibody against HIF-1α. PCR was employed to quantify the immunoprecipitated DNA and all values were normalized. The primer pairs for qPCR analysis were listed the Supplementary Table 4.

### Luciferase activity assay

786O cells were co-transfected with promoter firefly luciferase of target genes and plasmids of gene of interest using Lipofectamine Reagent (Invitrogen). Thirty-six hours later, luciferase activity was measured using the Dual-Luciferase Reporter Assay System (Promega) according to the manufacturer’s protocol. Luciferase activity was normalized to Renilla luciferase activity. All plasmid sources were listed the Supplementary Table 4.

### Cell viability detection

The cell counting kit-8 (CCK-8) assay was performed to observe the cell proliferation rate. 96-well plates were added 10 µl CCK-8 reagent (Dojindo Laboratories, Japan), then oscillated 2–5 min and finally detected OD value at 450 nm. For colony formation assay, fix cells for 30–60 min with 1 ml 4 % polyformaldehyde (Sinopharm Chemical Reagent Co., Ltd) per well. Dye the cells for 10–20 min with 1000 µl crystalline UV (Sangon Biotech Co,. Ltd). For EdU cell proliferation assay, cell culture medium was diluted EdU resolution with 1:1000 proportion. Fix the cells with 50 µl PBS containing 4 % paraformaldehyde. Dye the cells one by one using 1X Apollo 100 µl and 1X Hoechest 33,342 100 µl. Then count the decolorated cells. We also utilized crystal violet dye for detecting cell proliferation. Each experimental subgroup was repeated in triplicate.

### Cell cycle and apoptosis detection

For cell cycle assay, the cell suspension was washed and seeded onto 6-well plate, with 2 ml per well. Then cell staining solution was added for dying. For apoptosis detection, cells were stained with annexin V-PAC 10 µl, and PI 5 µl for 10–15 min (Thermo Fisher Scientific). Flow cytometry was used to detect and analyze the result.

### Migration and invasion assay

For transwell migration assay, with polycarbonate membrane as separation, DMEM and 10 % FBS served as nutrient solution in outer chamber, while 7 × 10^4 tumor cells per well were put into the inner chamber. After 16 h, the migrated cells were stained by crystal violet and counted by microscope. For invasion assay, transwell inserts (Costar) coated with Matrigel (BD Biosciences)/fibronectin (BD Biosciences) was utilized. To perform the wound healing assay, cell suspension was cultured 16–24 h as monolayer cells. Scratch the cells with the head of pipetting gun and add the 5-Fu solution incubating for 24 h, and change the 10 % FBS for 24 h. Use inverted microscope for observation and photo.

### Establishment of xenograft nude mice model and tail vein injection model

Tumor cells were cultured in DMEM medium. Extract 100 µl of the mixed cell (1 × 10^7^) suspension with 1 ml syringe and inoculated subcutaneously (s.c) on the right hind limb of the right back of the nude mice. On the 12th day of inoculation, the tumor volume of all nude mice was > 100mm^3^. The spirit, diet, defecation and activity of the nude mice were observed daily. The 5MPN or Sunitinib were added in the 14th day with doses indicated in the figure legends. The mass of the transplanted tumor was weighed and the long diameter (A) and short diameter (B) of the transplanted tumor were measured with a vernier caliper every 3 days from the 3rd day of inoculation. The mean volume of the transplanted tumor was calculated according to the volume formula V = 1/2 (A×B^2^), and the average value was obtained and the curve of tumor growth was plotted. On the 60th day after inoculation, the nude mice were sacrificed and the tumor was removed, and the morphology, texture and activity of the transplanted tumor were observed. For tail vein injection, 1 × 10^6^ of the prepared stable clones of Luc-labeled 786O cells suspended in 100 µL PBS were injected into the caudal vein to establish a model of renal cell carcinoma metastasis in nude mice. After 10 days of injection, lung tissue was taken for in vivo imaging to detect lung metastasis. At endpoint of another 4 weeks later, luciferase activity was measured again and relative change was compared.

### Statistical analyses

Statistical analysis for in silico studies were automatically performed with the platforms used, as aforementioned. Statistical analysis for in vitro assays and in vivo experiments were performed using the Prism Graphpad 9.0 for Mac. All assays were performed in triplicates. Comparisons between two groups were studied using the Mann-Whitney test for non-parametric variants and using the Student’s t test for parametric variants. IC50 for drug treatment was interpolated and fitted with sigmoidal curve. The survival data was presented using the Kaplan-Meier curve and compared using the Log-rank test. The *P* value of < 0.05 was accepted as significant [23].

## Results

### PFKFB4 is amongst the pivotal kinases in ccRCC

**Fig. 1:**
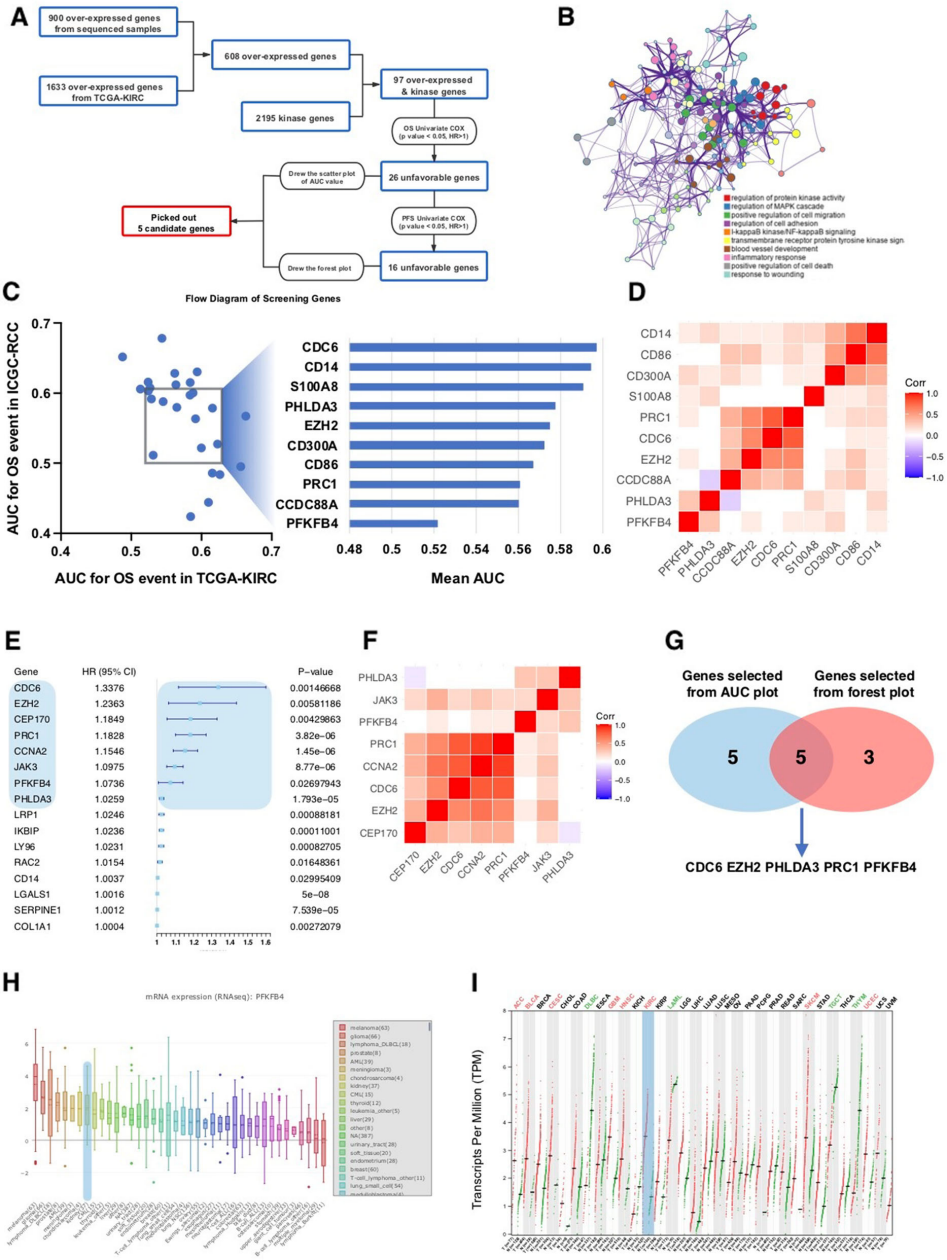
PFKFB4 is amongst the pivotal kinases in ccRCC. **A**) Workflow of candidate gene selection in the current study and derived from which was **B**) Functional network of kinase genes enriched in the current study; **C**) Plotting of area under curve (AUC) for overall survival (OS) in the Cancer Genome Atlas (TCGA) clear-cell renal cell carcinoma (KIRC) dataset against that in International Cancer Genome Consortium (IGCG) ccRCC dataset; **D**) Heatmap showing expressional correlations between candidate genes in (**C**); **E**) Forest plot of hazard ratio (HR) of candidate genes on progression-free survival; **F**) Heatmap showing expressional correlations between candidate genes in (**E**); **G**) Venn diagram of common genes from (**D**) and (**F**); **H**) Reproduced from TCGA dataset, shown was PFKFB4 expression in cancers with ccRCC highlighted; **I**) Reproduced from TCGA dataset, shown was deferential expression of PFKFB4 in paired normal and cancerous tissues with ccRCC highlighted

Kinases were of great interest in cancer research as they were feasibly drugable and tyrosine kinase inhibitors (TKIs) were the mainstay of systemic treatment of ccRCC. Using our microarray data in paired ccRCC tissue in combination with TCGA cohort we developed a workflow to identify candidates of pivotal kinase genes (Fig. 1 A). We first identified 608 commonly over-expressed genes among which 97 encoded kinase. Expressions of kinase genes in ccRCC participated in a variety of critical biologic processes besides kinase activity in ccRCC including angiogenesis, inflammation, etc. (Fig. 1B). We then applied univariate Cox exam for overall survival (OS) and identified 26 unfavorable genes. We first slelected top 10 prognostic kinase genes close to diagonal line in the AUC for OS event in both TCGA and IGCG cohorts (Fig. 1 C) with heatmap showing co-expressions (Fig. 1D). Among the unfavorable genes 16 were additionally associated with progression-free survival (PFS). Using the similar strategy, we selected top 7 candidates from the forest plot (Fig. 1E) with heatmap showing co-expressions (Fig. 1 F). Cross-referencing of the two sets generated 5 candidate genes (Fig. 1G). Co-expression of the genes (Fig. 1D F) showed that PFKFB4 and PHLDA3 were expressed relatively independently from other kinases (Fig. 1 F). Between the two only PFKFB4 was currently drugable with available compound of 5-(n-(8-methoxy-4-quinolyl)amino)pentyl nitrate (5MPN) and therefore became gene of interest in the current study. Of note, PFKFB4 expression in ccRCC was among the top 10 highest amid all TCGA cancers (Fig. 1 H). PFKFB4 was also differentially expressed in ccRCC with contrasting difference of expression between normal and cancer samples amongst all cancers (Fig. 1 I). Here we showed in silico that PFKFB4 could play pivotal role in ccRCC.

### PFKFB4 is differentially expressed in ccRCC

**Fig. 2:**
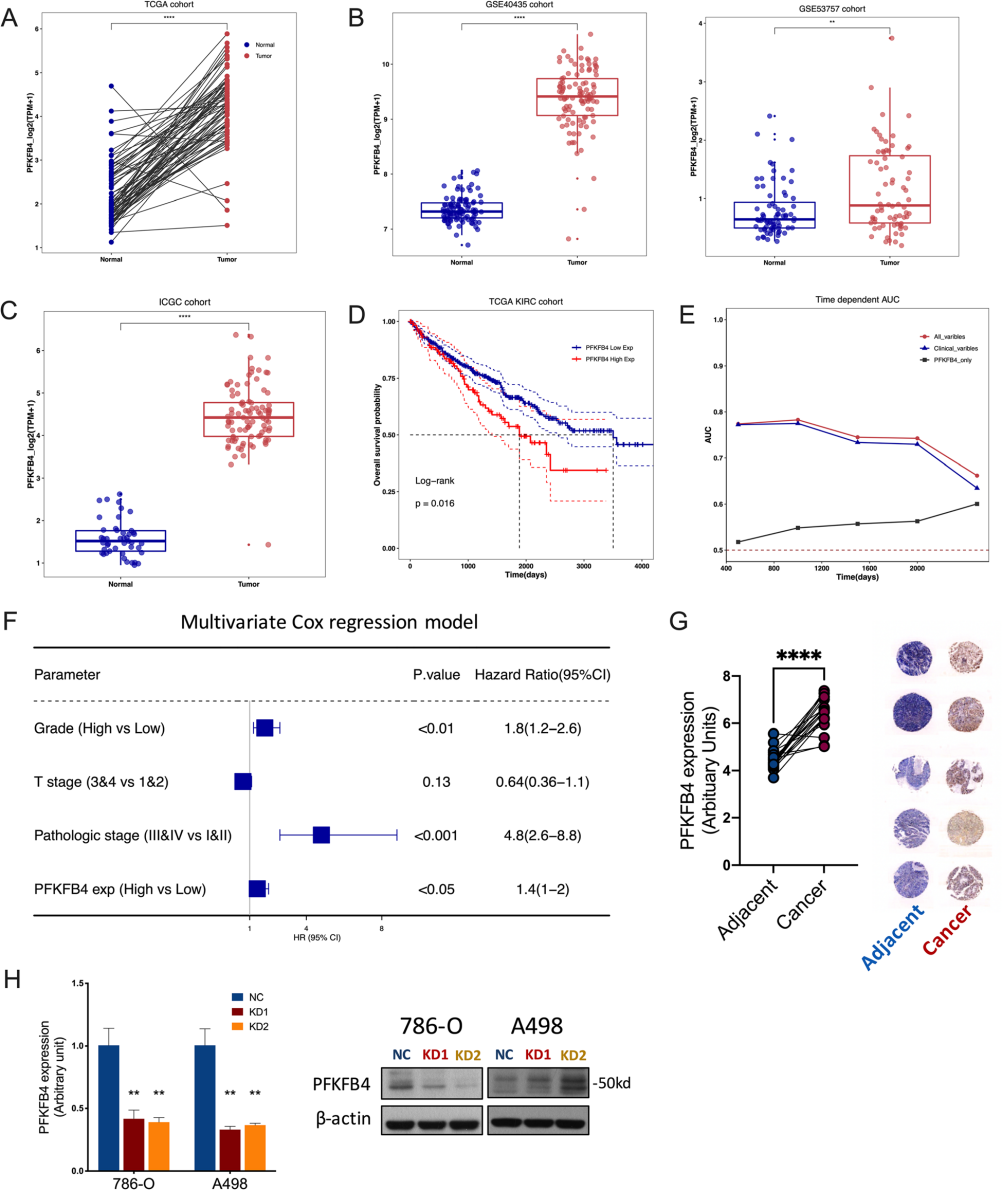
PFKFB4 is differentially expressed in ccRCC. **A**) Reproduced from the Cancer Genome Atlas (TCGA) clear-cell renal cell carcinoma (KIRC) dataset, shown was expression of PFKFB4 in paired normal and cancerous tissue of ccRCC, paired Student’s t test; **B**) Reproduced from 2 GEO datasets, shown were differential expressions of PFKFB4 in normal and ccRCC tissue, unpaired Mann-Whitney tests; **C**) Reproduced from International Cancer Genome Consortium (IGCG) ccRCC dataset, shown was differential expression of PFKFB4 in normal and ccRCC tissue, unpaired Mann-Whitney test; **D**) Kaplan-Meier plot of overall survival in TCGA-KIRC cohort grouped by higher and lower PFKFB4 expression, Log-rank test; **E**) The time-dependent receiver operating characteristic (ROC) analysis for the tumor grades, pathological stages, and the PFKFB4 levels in the TCGA-KIRC cohort; **F**) Multivariate analyses of TCGA-KIRC cohort with bars representing 95 % CIs; **G**) Retrieved from mRNA microarray data, shown was expression of PFKFB4 in 19 paired ccRCC samples (left panel) and representative immunohistochemical staining of PFKFB4 in 5 paired samples zoomed out to demonstrate expression trends (right panel), paired Student’s t-test. **H**) Efficacy of PFKFB4 knockdown (KD) in 2 ccRCC cell lines using 2 shRNAs (KD1 and KD2) and scrambled negative control (NC), measured by both quantitative PCR and western blotting. (*P < 0.05; **P < 0.01; ***P < 0.001; ****P < 0.0001)

To validate the findings, we queried PFKFB4 expression in paired kidney samples in TCGA cohort and found significantly higher expression in tumor tissue (Fig. 2 A). External validation in 2 independent GEO datasets (Fig. 2B) and the IGCG cohort (Fig. 2 C) also corroborated the differential expression of PFKFB4 in ccRCC. Higher PFKFB4 expression conferred significantly worsened overall survival (Fig. 2D) and the prognostic impact increased overtime in comparison to clinicopathological parameters of stage and grade (Fig. 2E). The Cox regression model showed that higher tumor grade, advanced pathological stage and higher PFKFB4 expression were independent prognostic factors, respectively (Fig. 2 F). In the 19 paired ccRCC samples that underwent microarray, we not only observed significantly overexpressed PFKFB4 but also detected substantial increased protein level of PFKFB4 in tumor than in adjacent kidney tissue (Fig. 2G). In a further IHC validation using 324 primary ccRCC sections, we found PFKFB4 expression significantly associated with older age, advanced tumor stage, grade and Ki-67 index (Table 1). Together, we showed that PFKFB4 was overexpressed in ccRCC tissue and tumors with higher PFKFB4 expression further demonstrated aggressiveness.

**Table 1:** Association between IHC score of PFKFB4, clinicopathological parameters and NCOA3 expression (SE = standard error)

**Parameter**	**Breakdown**	**N**	**PFKFB4 Expression**		**P**
			**Median**	**SE**	
**T**	T1-T2	258	1	0.04	<0.001
	T3-T4	66	2	0.12	
**N**	N0	274	1	0.04	0.003
	N1	50	2	0.14	
**M**	M0	314	1	0.04	<0.001
	M1	10	2	0.15	
**Gender**	Male	196	1	0.06	0.547
	Female	128	1	0.07	
**Grade**	I-II	262	1	0.04	<0.001
	III-IV	62	2	0.12	
**Neoadjuvant Tx**	No	306	1	0.04	0.001
	Yes	18	2	0.21	
**Correlation**		**Median**	**SE**	**Spearman r**	**P**
	**Age**	57	0.350	-0.449	<0.001
	**NCOA3**	1	0.040	0.810	<0.001

### PFKFB4-knockdown (KD) impairs cell fitness in ccRCC

**Fig. 3:**
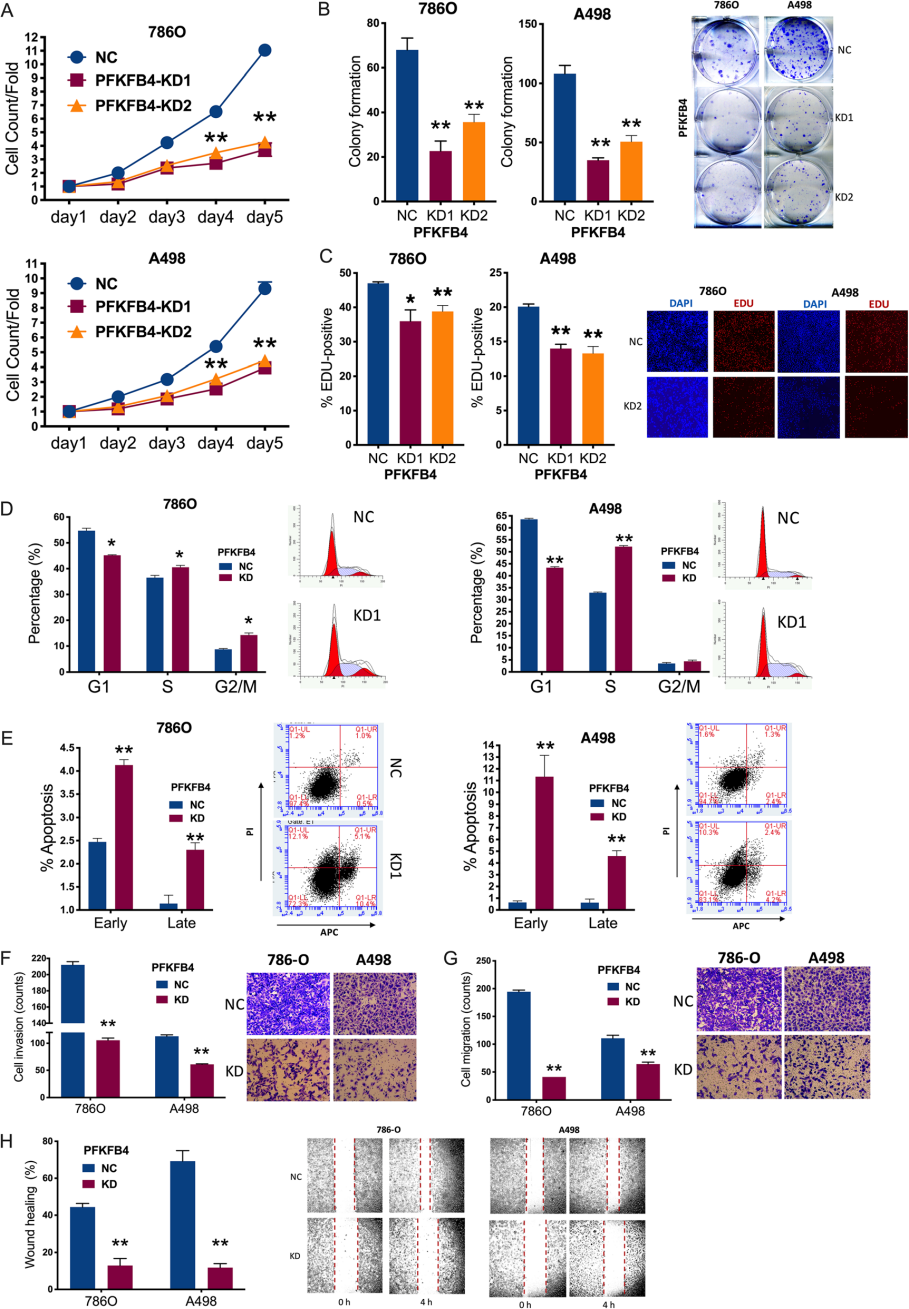
PFKFB4-knockdown (KD) impairs cell fitness in ccRCC. **A**) Cell count detected using CCK-8 in clear-cell renal cell carcinoma (ccRCC) cell lines with PFKFB4-knockdown (KD) by shRNA#1 and shRNA#2 (KD1 and KD2) or scrambled negative control (NC); **B**) Colony formation in ccRCC cell lines with PFKFB4 silencing or control); **C**) Proliferation detected using ratio of EDU/DAPI staining in ccRCC cell lines with PFKFB4-KD or NC; Flow cytometry used to detect **D**) cell cycle profile and **E**) apoptosis in ccRCC cells with PFKFB4-KD or NC; Transwell assays used to detect **F**) cell invasion with Matrigel and **G**) cell migration without Matrigel in ccRCC cells with PFKFB4-KD or NC; Wound healing assay in ccRCC cells with PFKFB4-KD or NC. (All in vitro assays performed in triplicates and at least 3 biological replicates; All comparisons by Student’s test, *N* = 5; ns = not significant; *P < 0.05; **P < 0.01; ***P < 0.001; ****P < 0.0001)

We next investigated role of PFKFB4 in vitro using 2 shRNAs targeting PFKFB4 in 2 ccRCC cell lines (Fig. 2 H). PFKFB4-KD significantly decreased cell proliferation in both cell lines (Fig. 3 A). PFKFB4-KD also significantly decreased colony formation in both cell lines (Fig. 3B). Both shRNAs significantly decreased EDU/DAPI ratio in both cell lines (Fig. 3 C). PFKFB4-KD significantly decreased cell population in G1 phase and increased population in G2 and M phase in 786O cells (Fig. 3D). Whereas alteration in G1 and G2 phases remained same in A498 cells, PFKFB4-KD did not alter population in M phase (Fig. 3D). Notably, flow cytometry showed PFKFB4-KD significantly induced both early and late apoptosis in both ccRCC cell lines (Fig. 3E). Transwell assays showed that PFKFB4-KD significantly decreased abilities in invasion (Fig. 3 F) and migration (Fig. 3G). Likewise, PFKFB4-KD resulted in delayed wound healing in both cell lines (Fig. 3 F). Here, we showed that PFKFB4-KD could substantially decrease fitness of ccRCC in vitro. we next sought to investigate the regulatory axis of PFKFB4.

### PFKFB4 is transcribed by HIF-1α in ccRCC

**Fig. 4:**
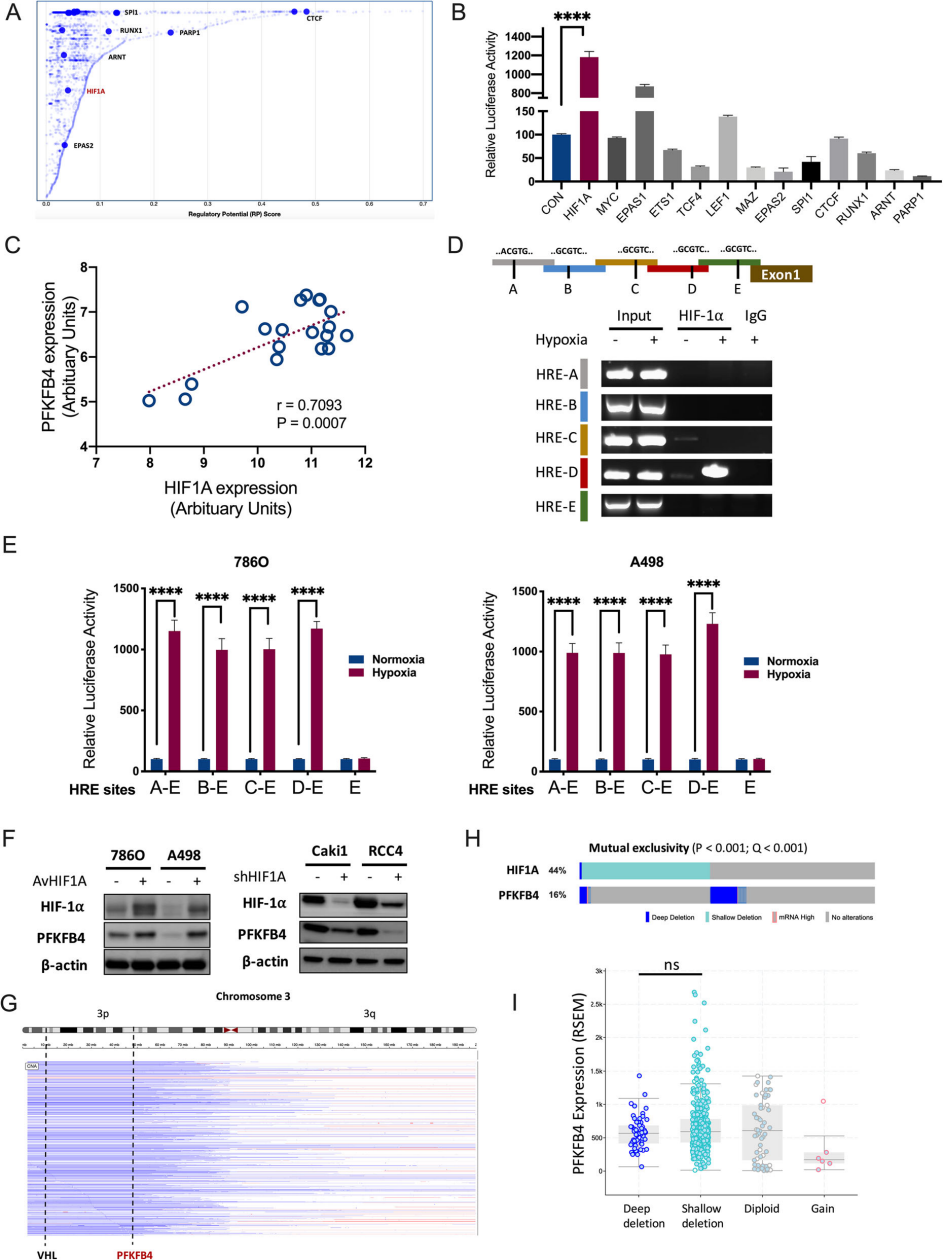
PFKFB4 is transcribed by HIF-1α in ccRCC. **A**) Reproduced from Cistrome ChIP-Seq dataset, shown were predicted transcription factors that could bind promoter of PFKFB4 in kidney tissue; **B**) Relative firefly-luciferase activity of PFKFB4 in 786O cells where Firefly luciferase activity was normalized to Renilla luciferase activity for all samples to yield relative luciferase activity, Student’s t-test; **C**) Reproduced from the Cancer Genome Atlas (TCGA) clear-cell renal cell carcinoma (KIRC) dataset, shown was expression correlation between PFKFB4 and HIF1A in ccRCC samples measure in microarray platform, Pearson correlation test; **D**) ChIP-PCR analysis of Flag marks (HIF1A) at the PFKFB4 promoter region in 786O cells with schematic diagram of PFKFB4 promoter regions and mouse IgG serving as negative control; **E**) HIF-1α binding to HRE-D (-270 ~ -290) site in the PFKFB4 promoter under the hypoxic condition in 786O cells as determined by ChIP assays under the hypoxic or normoxic conditions for 36 h before assays, with amount of DNA fragments pulled-down determined by real-time PCR; **F**) Western blotting showing HIF-1α and PFKFB4 level in 4 ccRCC cell lines with different basal HIF-1α level with knockdown (sh) or adenoviral overexpression (Av) of HIF1A; Reproduced from TCGA-KIRC dataset, shown were **G**) Genomic alteration of 3p and location of PFKFB4 in relation to VHL in ccRCC; **H**) Oncoprint of genetic alterations of HIF1A and PFKFB4 with mutual exclusivity detected by Chi-square test; **I**) mRNA expression of PFKFB4 against its copy number in ccRCC, Student’s t-test. (All in vitro assays performed in triplicates and at least 3 biological replicates; ns = not significant; *P < 0.05; **P < 0.01; ***P < 0.001; ****P < 0.0001)

We then set off to identify upstream regulator of PFKFB4. Through in silico analyses of ChIP-seq dataset Cistrome we identified 7 candidate TFs among which HIF1A was reported to transcribe PFKFB4 in other cancers [24] but was not validated in kidney cancer (Fig. 4 A). We thus conducted luciferase assay for candidate TFs both in Cistrome and Harmonizome and showed that HIF1A presented strongest activity in 786O cells (Fig. 4B). Expressions of HIF1A and PFKFB4 showed moderate to strong linear correlation in TCGA cohort (Fig. 4 C). ChIP-PCR showed that HIF-1α bound to HRE of PFKFB4 in 786O cells (Fig. 4D). The transcription activity was enhanced upon hypoxia within the corresponding binding site in both cell lines (Fig. 4E). Given that constitutive HIF1A expression varied drastically amid ccRCC cells, we overexpressed HIF1A in 786O and A498 cells with low basal HIF-1α level, and silenced HIF1A expression in Caki1 and RCC4 cells with high basal HIF-1α level. We found that PFKFB4 level corresponded to HIF-1α level regardless of cell type (Fig. 4 F). Intriguingly, PFKFB4 was located on 3p and HIF1A was located on 14q, both of which were recurrently deleted in ccRCC (Fig. 4G). We thus queried copy number alteration of PFKFB4 and HIF1A in TCGA cohort and found that deletion of the genes showed significant mutual exclusivity (Fig. 4 H), indicating functional necessity of retaining at least product from one gene. We also found that expression of PFKFB4 did not alter with change of copy number, further supporting the functional essentiality of PFKFB4 (Fig. 4I). Here, we showed that HIF-1α could be the upstream TF that activated PFKFB4 in ccRCC.

### PFKFB4 is associated with pentose phosphate pathway (PPP) in ccRCC

**Fig. 5:**
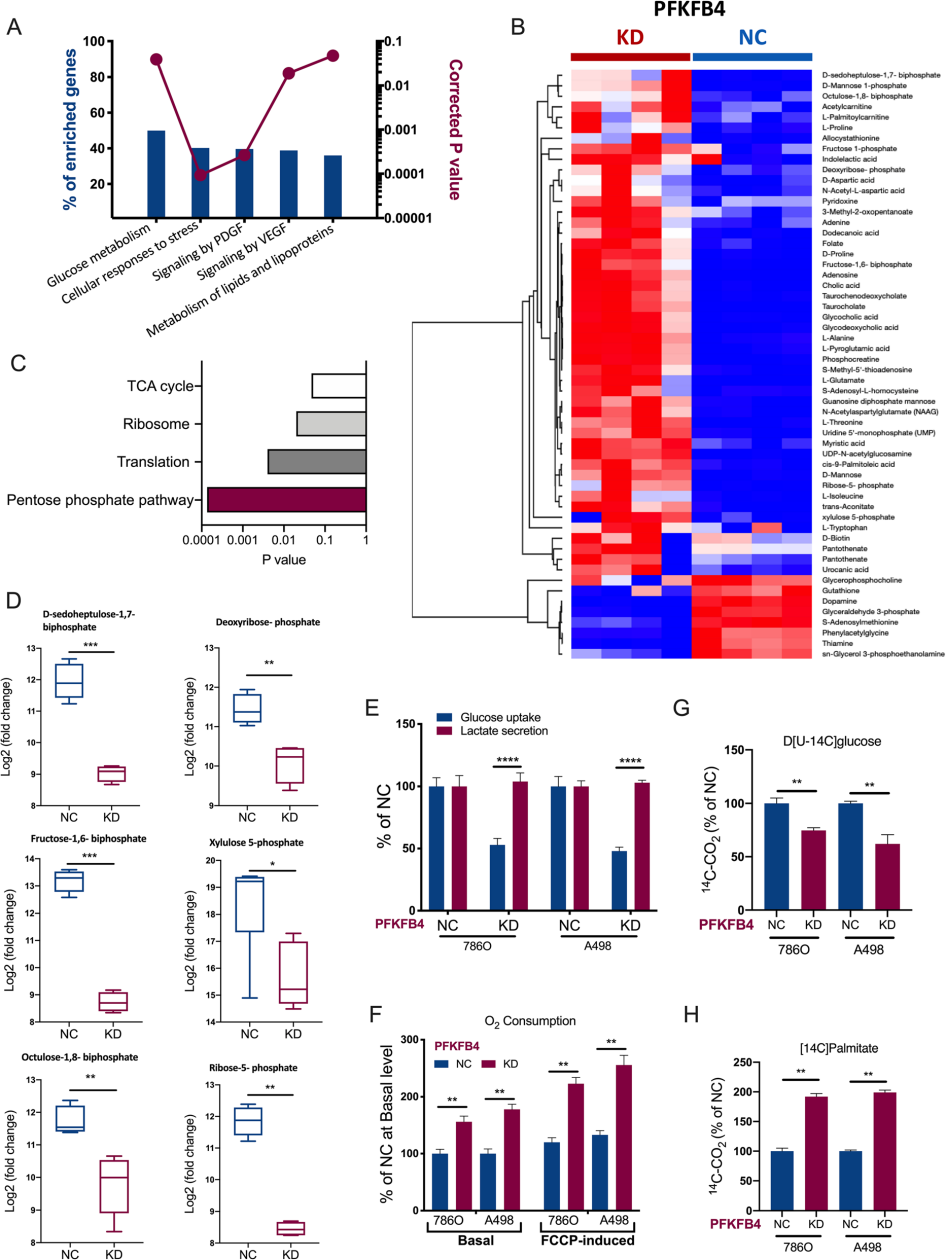
PFKFB4 is associated with pentose phosphate pathway (PPP) in ccRCC. **A**) from the Cancer Genome Atlas (TCGA) clear-cell renal cell carcinoma (KIRC) dataset, shown was functional annotation of gene enriched in PFKFB4-overexpressed cases (z-score of > 2 in RNA-seq samples) analyzed using NET-GE; **B**) Heatmap showing metabolites significantly changed in 786O cells with PFKFB4-knockdown (KD) versus negative control (NC); **C**) Metabolic Set Enrichment Analysis (MSEA) of 786O cells with PFKFB4-KD over NC; **D**) Box plots of individual pentose phosphate pathway metabolites that were significantly changed in 786O cells with PFKFB4-KD or NC; **E**) Glucose uptake and lactate secretion measured in 786O cells with PFKFB4-KD or NC; **F**) Intact cellular respiration measured using live cell real-time metabolism monitoring under basal conditions or in the presence of FCCP in786O cells with PFKFB4-KD or NC; **G**) Glucose oxidation measured by ^14^ C-CO_2_ production in 786O cells with PFKFB4-KD or NC following 3-hour labeling with D[U-14 C]glucose; **H**) Fatty acid oxidation measured by ^14^ C-CO_2_ production in 786O cells with PFKFB4-KD or NC following 3-hour labeling with [U-14 C]palmitate. (All in vitro assays performed in triplicates and at least 3 biological replicates; Student’s t-test for all comparisons; *N* = 4; ns = not significant; *P < 0.05; **P < 0.01; ***P < 0.001; ****P < 0.0001)

PFKFB4 was reported to exert dual function in other cancers [14]. On one hand, as a kinase PFKFB4 could phosphorylate downstream substrates. On the other, as a metabolic gene PFKFB4 could rewire glucose metabolism. We first sought to explore metabolic output of PFKFB4 in ccRCC. PFKFB4 overexpressed cases (z-score of > 2 in RNA-seq) in TCGA cohort showed enriched genes in several major signaling of ccRCC, including glucose metabolism, VEGF/PDGF pathway and lipogenesis (Fig. 5 A). Metabolomics analysis showed PFKFB4-KD induced substantial decrease of a variety of metabolites (Fig. 5B) among which the PPP was most enriched (Fig. 5 C). Select representative metabolites of PPP were significantly decreased in 786O cells with PFKFB4-KD (Fig. 5D). PFKFB4-KD significantly decreased glucose uptake whereas having no effect in lactate secretion in both ccRCC cell lines (Fig. 5E). PFKFB4-KD resulted in increased oxygen consumption (Fig. 5 F). Further dissecting changes in oxidative utilization of individual nutrients by measuring ^14^ C-CO2 release from cells labeled for 3 h with D[U-^14^ C]glucose or [U-^14^ C]palmitate further corroborated the findings that PFKFB4-KD decreased glucose oxidation (Fig. 5G-H). Here, we showed PFKFB4 could regulate PPP as a downstream metabolic output in ccRCC.

### PFKFB4 phosphorylates NCOA3 in ccRCC

**Fig. 6:**
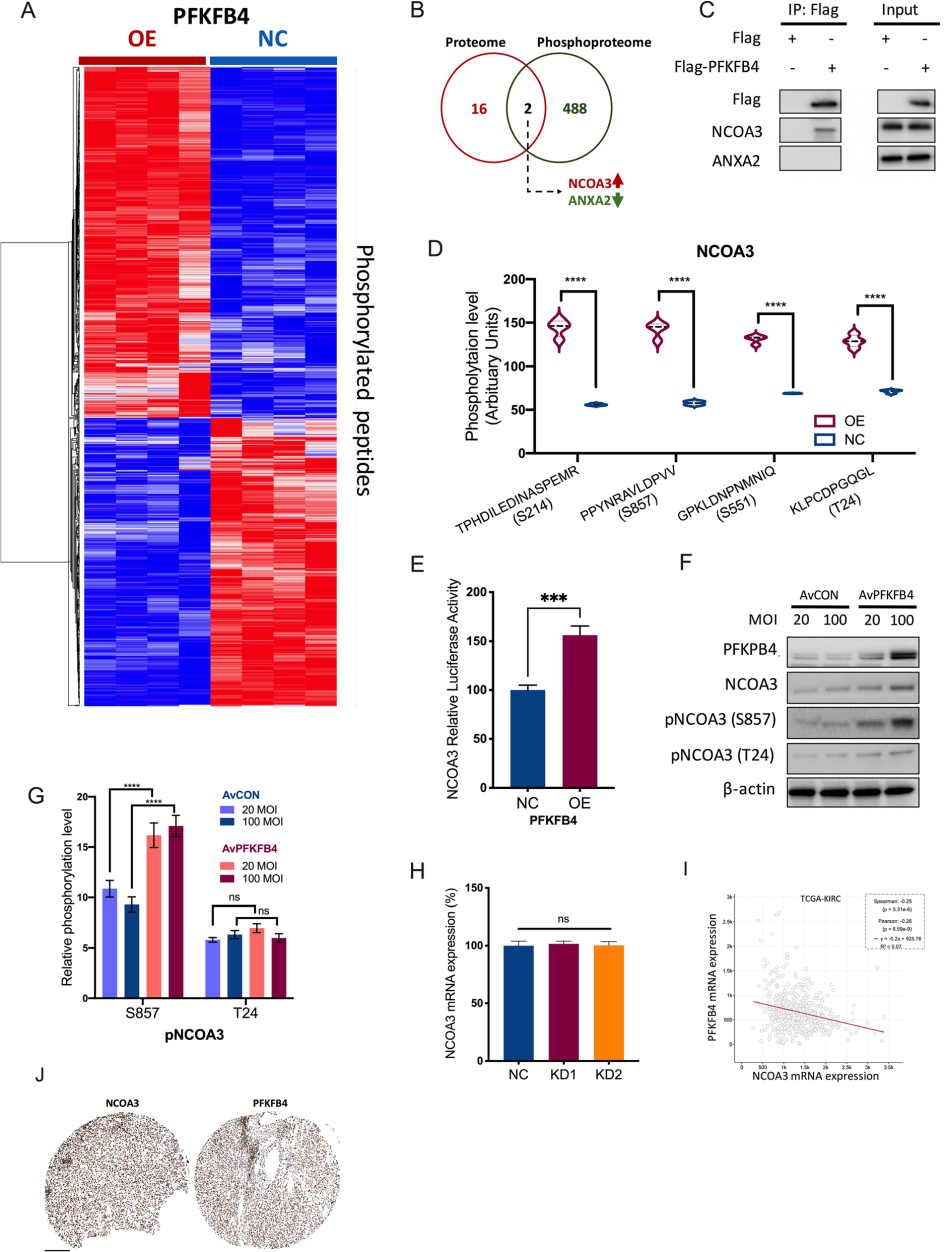
PFKFB4 phosphorylates NCOA3 in ccRCC. Phosphoproteomic assay using Tandem Mass Tag (TMT) technique in 786O cells with PFKFB4-knockdown (KD) versus negative control (NC) showing **A**) Clustering heatmap of deferentially phosphorylated peptides; **B**) Venn diagram of showing common proteins both significantly phosphorylated in phosphoproteomics and shown to interact with PFKFB4 in mass spectrum with **C**) validation using co-immunoprecipitation assay in lentiviral PFKFB4-overexpressed 786O cells; **D**) Violin plots of phosphorylation at each site of NCOA3 detected in the phosphoproteomics, Student’s t-test; **E**) Promoter luciferase assay showing activity of NCOA3 in 786O cells with PFKFB4-KD or NC, Student’s t-test; **F**) Western blotting showing phosphorylation levels at 2 sites of NCOA3 by different doses of adenoviral (Av) PFKFB4 overexpression and **G**) ratio of Phospho-NCOA3 over total NCOA3 measured by densitometry, two-way ANOVA; **H**) mRNA level of NCOA3 detected by q-PCR in 786O cells with PFKFB4-KD (2 shRNAs) or NC, one-way ANOVA; **I**) Reproduced from the Cancer Genome Atlas (TCGA) clear-cell renal cell carcinoma (KIRC) dataset, shown was expression correlation between PFKFB4 and NCOA3 at RNA-seq platform, both Pearson and Spearman correlations listed; **J**) Representative immunohistochemical staining of PFKFB4 and NCOA3 in the same ccRCC sample, bar = 200 μm. (All in vitro assays performed in triplicates and at least 3 biological replicates; ns = not significant; *P < 0.05; **P < 0.01; ***P < 0.001; ****P < 0.0001)

PFKFB4 was reported to phosphorylate SRC-3 (NCOA3) at Ser857 in breast cancer with no other report on its potential substrate [25]. To better understand the kinase activity of PFKFB4 in ccRCC, we performed phosphoproteomics to identify candidate substrate(s). Given the gain-of-function nature of PFKFB4 in ccRCC, we examined phosphopeptides using an overexpression (OE) model. The TMT assay generated a variety of significantly enriched phosphopeptides between PFKFB4-OE and control 786O cells (Fig. 6 A). Meanwhile, an IP-MS assay was performed to identify candidate protein(s) that co-precipitated with PFKFB4 and the intersection encompassed 2 proteins, NCOA3 (increased phosphorylation) and ANXA2 (decreased phosphorylation) (Fig. 6B). Co-IP further validated that only NCOA3 could be precipitated by PFKFB4 in 786O cells (Fig. 6 C). Mining of the phosphoproteomics showed that 4 peptides of NCOA3 were included in the assay and all were significantly phosphorylated in our study, including the previously reported S857 (Fig. 6D). Overexpression of PFKFB4 increased transcriptional activity of NCOA3 in 786O cells (Fig. 6E). As only antibodies against S875 and T24 were commercially available and we have been thus far unsuccessful developing antibodies against S214 and S551, we validated that phosphorylation of both S875 and T24 sites were increased in a dose-dependent manner following PFKFB4-OE (Fig. 6 F). Though total protein of NCOA3 was also increased, as previously reported, the ratio of p-NOCA3 remained significantly increased at both T24 and S857 (Fig. 6G). Interestingly, mRNA level of NCOA3 did not alter following PFKFB4-KD in 786O cells (Fig. 6 H). Overall, NCOA3 expression even demonstrated a weakly negative linear correlation with PFKFB4 expression, further indicating that regulation of NCOA3 could be promiscuous in ccRCC in which PFKFB4 played a role in part (Fig. 6I). Also, we validated the correlation between total protein levels of NCOA3 and PFKFB4 by IHC in our in-house ccRCC samples showing a positive correlation therein (Fig. 6 J, Table 1). Here, we showed PFKFB4 demonstrated kinase activity by interacting with and phosphorylating NCOA3 in ccRCC.

### PFKFB4-NCOA3-FBP1 forms regulatory loop

**Fig. 7:**
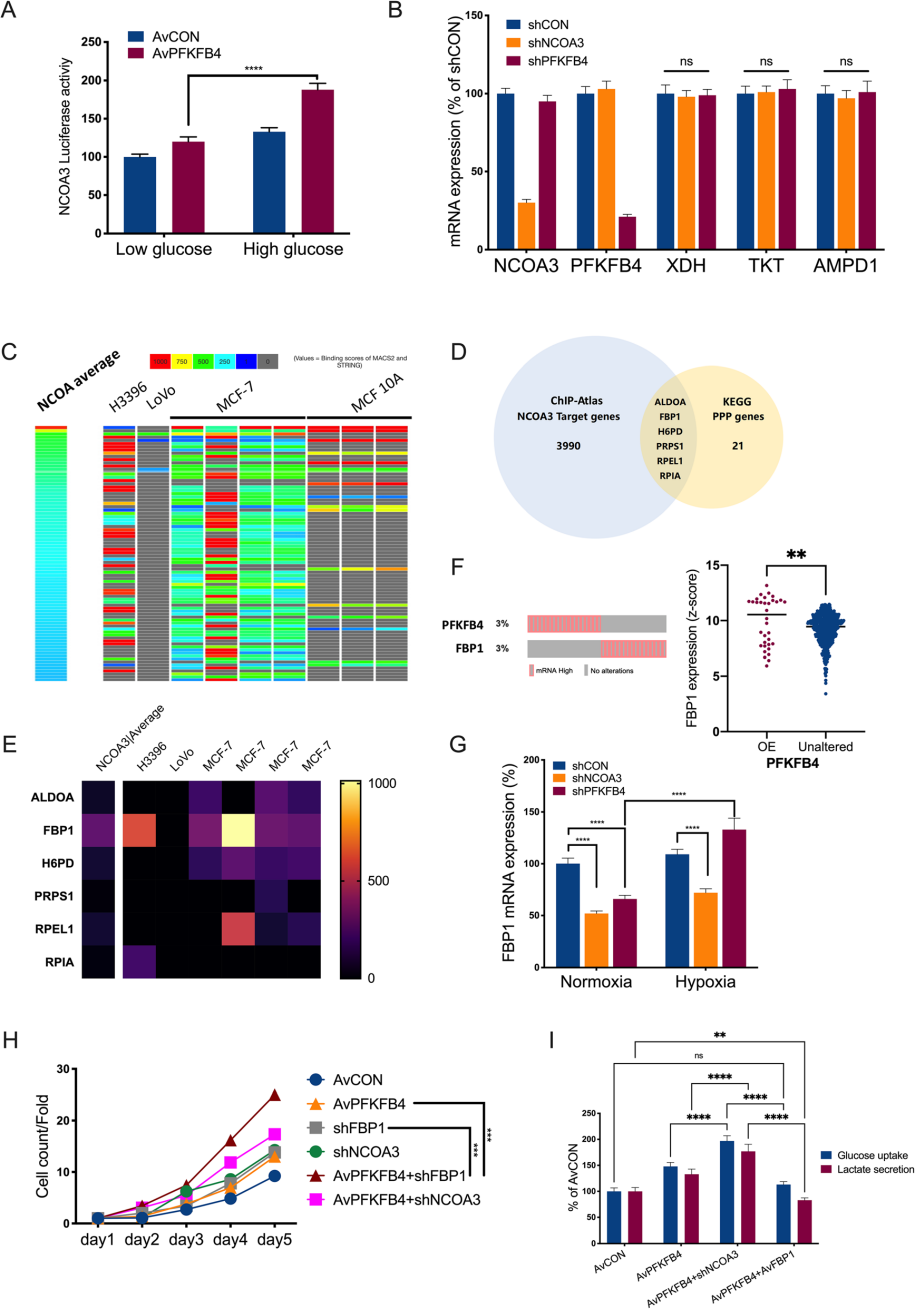
PFKFB4-NCOA3-FBP1 forms regulatory loop. **A**) Luciferase activity of NCOA3 in 786O cells with adenoviral (Av) PFKFB4 overexpression upon high (25 mM) and low (5 mM) glucose culture, two-way ANOVA; **B**) Q-PCR showing mRNA expression of target genes and 3 pentose phosphate pathway (PPP) genes upon knockdown of PFKFB4 or NCOA3 with shRNAs, one-way ANOVA; **C**) Reproduced from ChIP-Atlas, shown was heatmap ranked by binding score of NCOA3 from high to low in different model cells, each row representing one gene; **D)** Venn diagram showing PPP genes that could bound NCOA3 shown in ChIP-Atlas and **E**) heatmap showing their binding scores; **F**) Reproduced from the Cancer Genome Atlas (TCGA) clear-cell renal cell carcinoma (KIRC) dataset, shown were OncoPrint of PFKFB4 and FBP1 expression of z-score > 2 by RNA-seq (left) and FBP1 expression in patients with or without PFKFB4 overexpression, with patients with FBP1 overexpression excluded (right), Student’s t-test; **G**) Q-PCR showing FBP1 expression in 786O cells with PFKFB4 or NCOA3 knockdown (shRNA) under different oxygen status, two-way ANOVA; **H**) Cell count detected using CCK-8 in 786O cells with overexpression and knockdown of indicated genes, two-way ANOVA; I) Glucose uptake and lactate secretion measured in 786O cells in 786O cells with overexpression and knockdown of indicated genes, two-way ANOVA. (All in vitro assays performed in triplicates and at least 3 biological replicates; ns = not significant; *P < 0.05; **P < 0.01; ***P < 0.001; ****P < 0.0001)

**Fig. 8:**
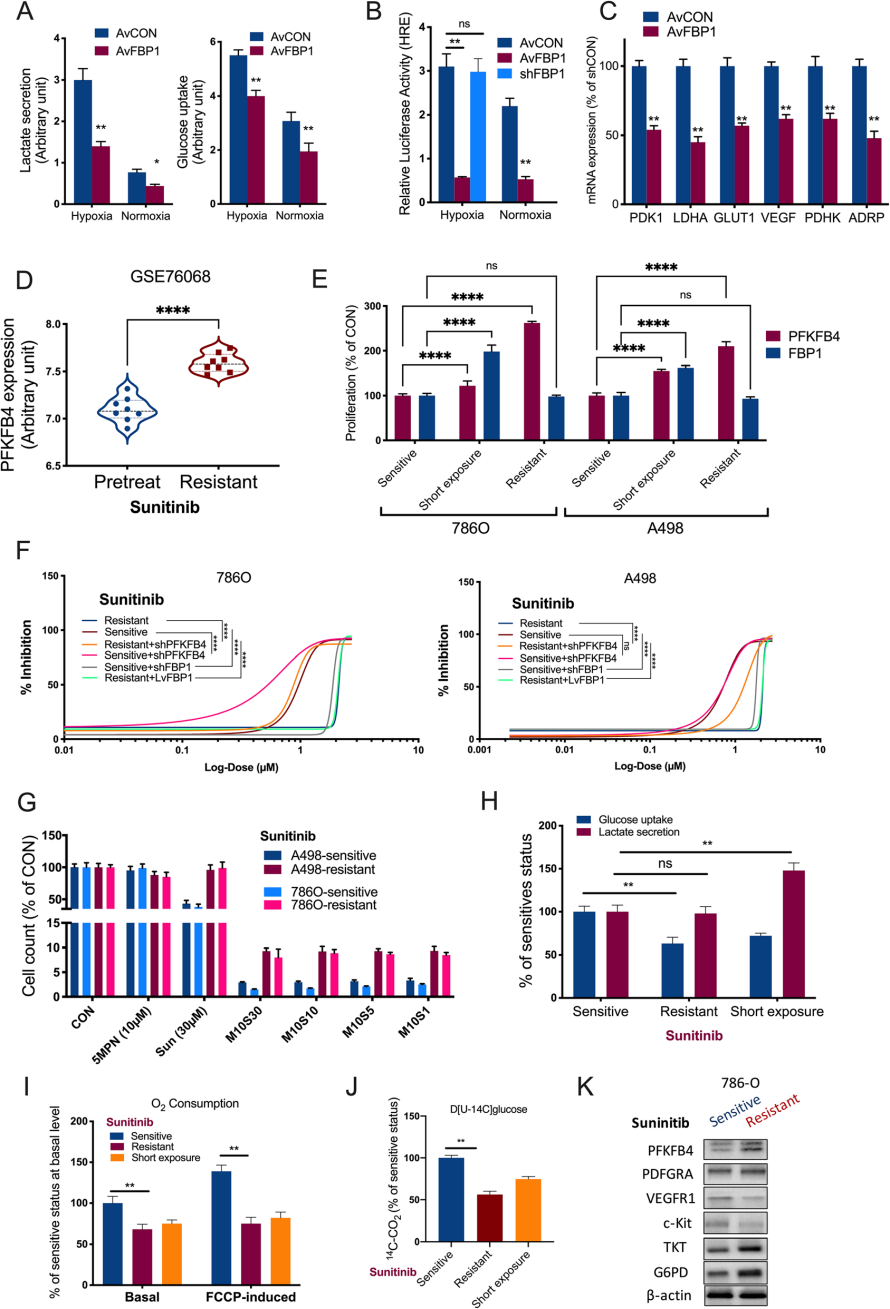
Pentose phosphate pathway (PPP) is associated with FBP1 and Sunitinib resistance in ccRCC. **A**) Glucose uptake and lactate secretion measured in 786O cells with FBP1 overexpression (AvFBP1) or control (AvCON) under hypoxia and normoxia; **B**) Relative firefly-luciferase activity of hypoxia response element (HRE) in 786O cells under hypoxia and normoxia in 786O cell with FBP1 overexpression, knockdown (shFBP1) or control; **C**) mRNA expression of target genes of HIF1A in 786O cells with FBP1 overexpression or control; **D**) Reproduced from in GEO dataset, shown was violin plot of PFKFB4 expression level in pretreat and Sunitinib (Sun)-resistant ccRCC, Student’s t-test; **E**) Proliferation detected by crystal violet assay in 3 ccRCC cell lines with different sensitivity to Sun harvested on day 3, with short exposure denoting sensitive cells being exposed to 30 µM of Sun for 72 h, two-way ANOVA; **F**) Shifting of sigmoidal dose-response fitting curve of Sun applied to 2 ccRCC cell lines with lentiviral overexpression (Lv) or knockdown (sh) of target genes; **G**) Proliferation detected using crystal violet at 72 h of treatment with 10 µM of 5MPN (M10) or combined with different doses (µM) of Sun (S30 to S1) in 2 ccRCC cell lines with different Sun sensitivity profile, all normalized to control (CON); Metabolic analysis in 786O cells with short Sunitinib exposure, Sunitinib –sensitive and -resistant 786O cells, shown were **H**) Glucose uptake and lactate secretion; **I**) Intact cellular respiration measured using live cell real-time metabolism monitoring under basal conditions or in the presence of FCCP; **J**) Glucose oxidation measured by ^14^ C-CO_2_ production following 3-hour labeling with D[U-14 C]glucose; **K**) Western blotting of select Sunitinib targets and PPP genes. (All in vitro assays performed in triplicates and at least 3 biological replicates; ns = not significant; *P < 0.05; **P < 0.01; ***P < 0.001; ****P < 0.0001)

Thus far, we have shown that gain-of-function of PFKFB4 enhanced PPP in ccRCC. However, unlike glycolysis PPP was not predominantly overactive in ccRCC, especially in treatment-naïve status, indicating PPP might not be the primary approach to hijack energy [26]. This notion contradicted in part with our finding with gain-of-function of PFKFB4 and we thus hypothesized that there could be signaling counteracting PFKFB4, fine-tuning the PPP activity. We first showed that high glucose could enhance NCOA3 activity, supporting its downstream regulation of glucose metabolism (Fig. 7 A). However, expressions of candidate PPP genes that changed with NCOA3 activity in breast cancer did not significantly alter in ccRCC (Fig. 7B). As NCOA3 was a transcription co-activator, we predicted its bind partner using ChIP-Atlas and ranked binding score of each candidate genes (Fig. 7 C). Among the 3990 candidate targets there were 6 PPP genes (Fig. 7D) and FBP1 had the highest binding score (Fig. 7E). Of note, FBP1 was reported to be a tumor suppressor constantly deleted in ccRCC that inhibited glycolysis and PPP with direct inhibition of HIF-1α activity, putting FBP1 in the opposite position to PFKFB4. Correspondingly, we observed mutually exclusive pattern of ccRCC cases with overexpression of PFKFB4 or FBP1 (Fig. 7 F). By excluding primary FBP1-OE cases, we found that FBP1 expression was significantly higher in PFKFB4-OE cases (Fig. 7 F). Under normoxia FBP1 expression was decreased with either NCOA3- or PFKFB4-KD, whereas under hypoxia FBP1 expression was significantly increased upon PFKFB4-KD, supporting the truncal effect of FBP1 as previously reported (Fig. 7G). PFKFB4-OE combined with FBP1-KD demonstrated potent protumorigenic effect compared with either modification alone (Fig. 7 H). Notably, overexpression of FBP1 completely restored alteration in glucose uptake and lactate secretion induced by PFKFB4-OE with or without NCOA3 silencing (Fig. 7I). FBP1 was shown to exert dual function in ccRCC by promoting gluconeogenesis counteracting PPP and glycolysis, and by inhibiting transcriptional activity of HIF[27]. We validated the findings in the current study using 786O cells. As expected, FBP1-OE significantly reduced glucose uptake and lactate secretion, both of which being intensified at hypoxia status in 786O cells which, of note, harbored relatively low constitutive HIF1A expression (Fig. 8 A). We then validated the HIF regulatory role of FBP1 and found FBP1-OE significantly inhibited Hypoxia Responsive Elements (HRE) in 786O cells (Fig. 8B). Consequently, expressions of a series of HIF1A target genes were significantly down-regulated (Fig. 8 C). We thus corroborated our hypothesis in part that PFKFB4 could induce FBP1 expression via NCOA3 phosphorylation as a negative feedback to curb PPP in ccRCC.

### PFKFB4-knockdown overcomes Sunitinib resistance in ccRCC

**Fig. 9:**
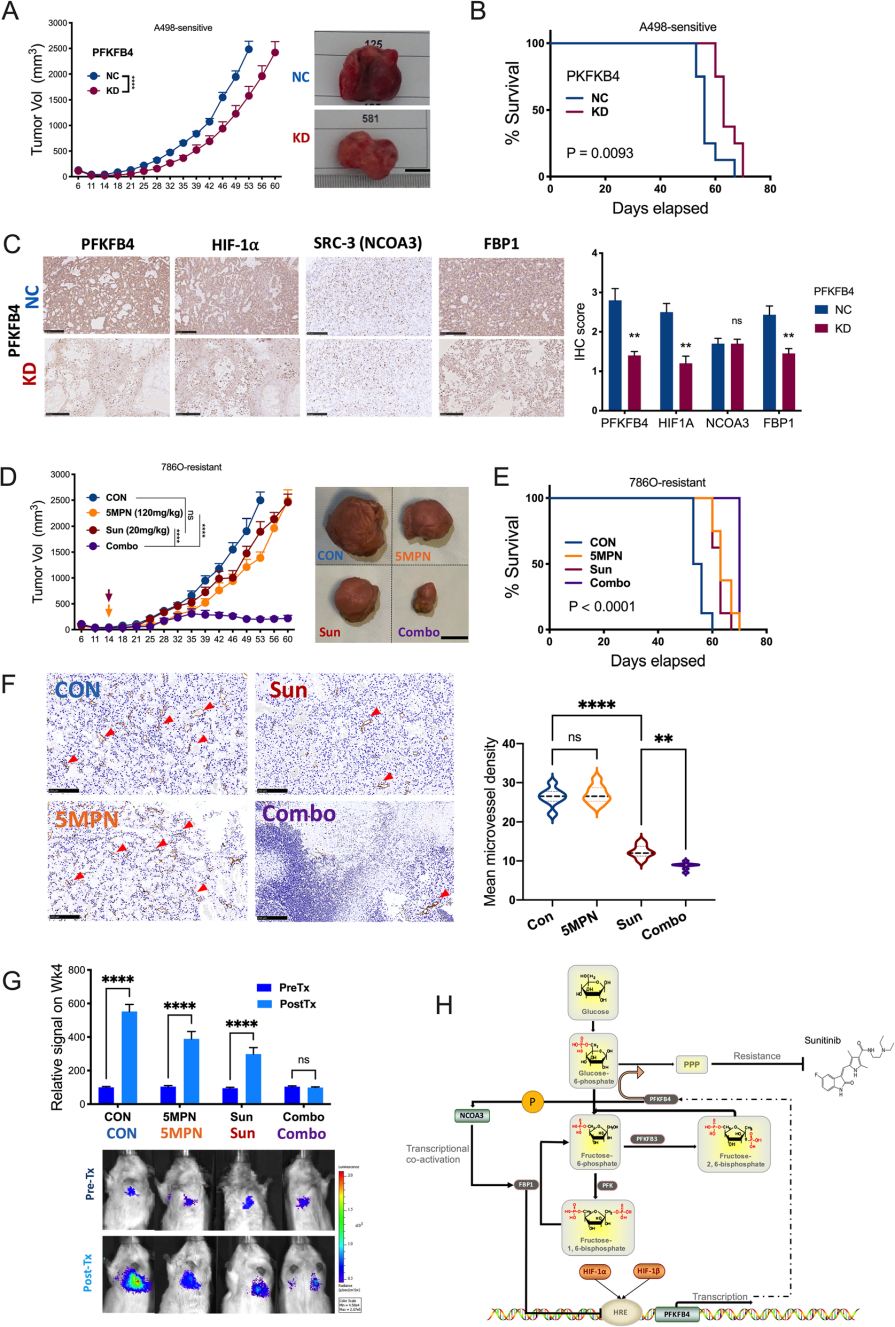
PFKFB4-knockdown overcomes Sunitinib resistance in ccRCC in vivo. **A**) Xenograft murine models consisting of 8 male BALB/c nude mice per group with subcutaneous implanted Sun-sensitive A498 cells with or without PFKFB4-KD (shRNA#2) under right hind limb with tumor growth monitored over 60-day period and tumor size of < 2500 mm^3^ as endpoint, with representative tumor image at endpoint (bar = 1 cm) analyzed by two-way ANOVA and **B**) Kaplan-Meier curves of survival of mice, analyzed by Log-rank test; **C**) Representative immunohistochemical staining of factors in tumors from A), with intensity scored semi-quantitatively and statistically compared; **D**) Xenograft murine models consisting of 8 male BALB/c nude mice per group with subcutaneous implanted Sun-resistant 786O cells fed with 20 mg/kg of Sun and/or 120 mg/kg of 5MPN orally by gavage; tumor growth monitored over 60-day period, analyzed by two-way ANOVA and **E**) Kaplan-Meier curves of survival of mice, analyzed by Log-rank test; **F**) Representative immunohistochemical staining CD31 targeting micro-vessels in tumors (red arrows) from D), with relative micro-vessel density (MVD) statistically compared; **G**) Tail vein injection of Sun-resistant A498 cells in 9 mice per group with 5MPN, Sun or combo treatments (Tx); mice monitored for 4 weeks for photon detection, normalized to PreTx CON group; bar figures showing photon change before (PreTx) and after (PostTx) with representative luciferase image showing lung involvement at endpoint of each group **H**) Schematic cartoon of regulatory axis proposed by the current study. (All in vitro assays performed in triplicates and at least 3 biological replicates; ns = not significant; *P < 0.05; **P < 0.01; ***P < 0.001; ****P < 0.0001)

PPP was shown to play a role in Sunitinib resistance in ccRCC[21]. We next examined whether PFKFB4 was associated with Sunitinib sensitivity. Reproduction of the GEO dataset (GSE76068) showed that Sunitinib-resistant ccRCC cells harbored significantly higher PFKFB4 expression (Fig. 8D). Interestingly, we showed that expressions of PFKFB4 and FBP1 was solely linked in the status of short Sunitinib exposure whereas in resistant status, overexpressed PFKFB4 was no longer inhibited by FBP1 which returned to initial level (Fig. 8E). IC50 assays showed that PFKFB4-KD significantly decreased IC50 of Sunitinib in resistant cell lines (Fig. 8E). FBP1-KD in sensitive cell lines also resulted in increased IC50, whereas FBP1-OE in resistant cells could not restore sensitivity (Fig. 8E). Whether sensitive cells with PFKFB4-KD could further reduce IC50 depended on cell context with positive result solely observed in 786O cells (Fig. 8E). Overview of drug sensitivity showed combination of 5MPN at 10 µM could reduce Suninitb dose to 1 µM with comparable effect to Sunitinib at 30 µM, indicating potent combination effect (Fig. 8G). Compared to sensitive status, both short exposure of Sunitinib and Sunitinib-resistant status showed increased lactate secretion and decreased glucose uptake (Fig. 8 H). Consistently, decreased oxygen consumption was observed in Sunitinib-treated 786O cells (Fig. 8I). Similar trend was also observed in CO_2_ generation in 3 cell lines (Fig. 8 J). As cells at short exposure of Sunitinib were under selection and were clonal heterogeneous, we compared expressions of Sunitnib targets and select PPP genes in Sunitnib -sensitive and –resistant 786O cells. We noted decreased levels of target receptor tyrosine kinase (RTK) VEGFR1 and c-Kit, and increased PPP enzyme TKT and G6PD (Fig. 8 K). Three in vivo models were used to validate findings in vitro. In the xenograft model with Sunitinib-sensitive cells, growth of PFKBP4-KD tumors was significantly slower than that of control tumors (Fig. 9 A). PFKBP4-KD also conferred significantly prolonged survival (Fig. 9B). PFKFB4-KD tumors showed significantly lower expressions of PFFB4, HIF1A and FBP1 whereas NCOA3 expression remained unchanged (Fig. 9 C). Xenograft models implanted with 786O cells were treated pharmaceutically and combination of 5MPN and Sunitinib showed potent synergy (Fig. 9D). Of note, possibly due to angiogenesis, pharmaceutical effect of monotherapy differed substantially from in vitro assays (Fig. 9D and E). By staining extracted tumors with CD31-labeled micro-vessel density (MVD), we observed significantly decreased MVD in Suninitib treated tumors (Fig. 9 F). As expected, group with combination therapy showed significantly prolonged survival (Fig. 9G). In the tail vein A498 injection model, combination treatment resulted in potent inhibition of pulmonary metastasis of tumor cells at endpoint of study (Fig. 9G). Interestingly, effect of monotherapy with Sunitinib was slightly superior to that of 5MPN (Fig. 9G). Together, we showed that genetic and pharmaceutical inhibition of PFKFB4 sensitized ccRCC to Sunitinib.

## Discussion

In the current study, we have shown that PFKFB4 plays a role not only in tumor development of ccRCC but also assist acquisition of Sunitinib-resistance phenotype. We depicted both HIF1A as a TF upstream and PPP and NCOA3/FBP1 as downstream output of PFKFB4 in ccRCC. Of note, many of the regulatory steps of PFKFB4 have been reported separately in individual cancers. We have thus based on our hypothesis by jigsawing many parts of evidence and conducted the current proof-of-concept study.

Loss of 3p in clear-cell carcinoma targets driver genes of VHL, PBRM1, SETD2 and BAP1 with collateral deletion of a series passenger genes, amongst which certain genes harbor indispensable functions to maintain cancer cell viability. With only limited copy number left, such genes should be upregulated at transcription level to compensate for decreased gene dosage. PFKFB4 is located on 3p21.31 and is deeply deleted in the majority of ccRCC samples. It is however upregulated and plays key role in regulating the concentration of the glycolytic byproduct fructose-2,6-bisphosphate (F2,6BP), and is usually induced highly expressed by hypoxia in tumors, indicating the critical role of the gene. PFKFB4-mediated glycolysis was associated with cancer stemness in breast cancer, while the inhibition of this protein may lead to improved outcome for patients [28]. Similarly, enhanced glycolysis during the androgen-independent growth of LNCaP-AI cell line and tumor progression were verified attributed to PFKFB4 overexpression in prostate cancer [29]. Moreover, the mRNA expression of PFKFB4 served as prognostic biomarker in solid tumors including breast cancer [26], bladder cancer [30] and non-small cell lung cancer [31]. Thus, PFKFB4 participating in core metabolic pathways have proven to be essential for the proliferation and survival of cancer cells. In accord with above cancers, we found that PFKFB4 was overexpressed in renal tumor cells which suggested worse prognosis, and it functioned as a regulator in metabolic programming to induce proliferation, migration and invasion of RCC.

NCOA3 (also known as SRC-3) has been described as oncogene in many studies. It was found overexpressed in 60 % breast cancer patients, leading to tamoxifen resistance and worse clinical outcome, while the NCOA3 deficiency could suppress the tumor initiation and progression in mice model with breast cancer [32]. Via regulating the telomerase reverse transcriptase (TERT) signaling, NCOA3 promoted cell viability and colony formation in hepatocellular carcinoma cells, and high expression of NCOA3 had worse prognosis [33]. Dusgupta et al [26] unveiled that PFKFB4 phosphorylated SRC-3(also known as NCOA3) to drive glucose flux towards the pentose phosphate pathway, demonstrating the correlation between metabolic reprogramming and transcriptional regulation. Similarly, we confirmed the interaction between NCOA3 and PFKFB4 to modulate PPP flux in renal cell carcinoma. Whereas silencing PFKFB4 showed potent inhibition in ccRCC cells regardless of HIF1A status, PFKFB4-OE did not per se promote tumor growth. We thus further studied clinicopathological associations of PFKFB4 and found its expression was associated with essential parameters like tumor stage, grade, nodal involvement or metastasis. Such findings were in strong agreement with its prognostic effect and differential expression. We thus further hypothesized that PFKFB4 expression was associated with therapeutic outcome, possibly response to tyrosine kinase inhibitors (TKIs) in the era when TCGA study was conducted.

Metabolic reprogramming is one of the hallmarks of cancer [34]. Metabolomic analysis showed distinct characteristics of enhanced intake and utilization of glucose in renal tumor cells, suggesting altered metabolic profile covering glycolysis and pentose phosphate pathway (PPP). Elevated levels of PPP-related metabolites including glucose-6-phosphate dehydrogenase (G6PDH) highlighted the importance of PPP in ccRCC [35]. When inhibiting G6PDH in renal tumor cells, decreased nicotinamide adenine dinucleotide phosphate (NADPH) level and increased level of ROS were observed, implying critical modulator of PPP in ccRCC redox homeostasis. Furthermore, high level of NADPH brought by activated PPP allowed resistance to apoptosis, oxidative stress, and radiation, which supported the rapid proliferation of ccRCC cells[36]. Besides, the increased expression of transketolase-like 1 (TLKL-1) protein, one of key enzymes involved in the PPP, predicted more malignant phenotype and facilitate the tumor growth especially in hypoxic condition. Notably, TLKL-1 may be associated with resistance to anti-angiogenesis targeted treatment[37]. Herein, we confirmed PFKFB4 phosphorylation as new modulator in PPP activities and reprograms the metabolism of ccRCC.

Notably, we found a negative regulatory loop involving PFKFB4/HI1A/FPB1 in ccRCC. The loop not only validated the reported role of FBP1 in ccRCC[27], but also explained in part why glycolysis is much more often reported in ccRCC rather than PPP. We speculate that basal PPP level is fine-tuned in part by the loop as it provides limited growth advantage compared with glycolysis. Activated PPP is suggested to buffer unexpected selection pressure such as prolonged drug treatment (i.e. Sunitinib) (Fig. 9 H). The regulation of PFKFB4 by HIF-1α also echoes the latest point of view that HIF-1α plays an oncogenic role at early and late stages of ccRCC development and progression. It is highly possible that PFKFB4 act as the failsafe to counteract excessive hypoxia-induced ROS incurred either by selective pressure or anti-cancer agents like Sunitinib. The fine-tuning between glycolysis and PPP by PFKFB4 may reflect the resilience of ccRCC to ever-changing micro-environment.

## Conclusions

PFKFB4 was overexpressed in ccRCC and was associated with aggressive phenotype and with PPP activity and the fine-tuning of which was mediated by its phosphorylation of NCOA3. NCOA3 interacted with FBP1 to counteract overactive PPP flux, forming a regulatory loop Targeting PFKFB4 held promise to combat resistance to Sunitinib.

## Supplementary information

**Additional file 1: Suppl Table 1.**

**Additional file 2: Suppl Table 2.**

**Additional file 3: Suppl Table 3.**

**Additional file 4: Suppl Table 4.**

## Data Availability

Three datasets were generated in the current study and were all submitted as the supplementary files.

## Abbreviations

ccRCC: clear-cell renal cell carcinoma
G6P: glucose 6-phosphate
HIF: hypoxia-inducible factor
PPP: pentose phosphate pathway
F6P: fructose 6-phosphate
F-1,6-2P: 1,6-bisphosphate
PFKFB: 6-phosphofructo-2-kinase/fructose-2,6-bisphosphatase
OS: overall survival
PFS: progress-free survival
AUC: area under curve
UHPLC: ultra-high-performance liquid chromatography
ESI: electrospray ionization
KEGG: Kyoto Encyclopedia of Genes and Genome
MSEA: metabolite Set Enrichment Analysis
TMT: tandem mass tag
FASP: filter aided proteome preparation
TKIs: tyrosine kinase inhibitors
NCOA3: Nuclear receptor coactivator
ANXA2: Annexin A2
FBP1: Fructose-Bisphosphatase 1
VHL: Von Hippel-Lindau
PBRM1: Polybromo 1
SETD2: SET Domain Containing 2
BAP1: BRCA1-Associated Protein 1
TERT: telomerase reverse transcriptase
NADPH: nicotinamide adenine dinucleotide phosphate
TLKL-1: transketolase-like 1
