# Correction to: PFKFB4 is overexpressed in clear-cell renal cell carcinoma promoting pentose phosphate pathway that mediates Sunitinib resistance

**DOI:** 10.1186/s13046-021-02165-5

**Published:** 2021-12-02

**Authors:** Chenchen Feng, Yuqing Li, Kunping Li, Yinfeng Lyu, Wenhui Zhu, Haowen Jiang, Hui Wen

**Affiliations:** 1 Department of Urology, Huashan Hospital, Fudan University, 12 Middle Urumqi Rd, 200040 Shanghai, PR China; 2 Institute of Urology, Fudan University, 200040 Shanghai, PR China

**Correction to: J Exp Clin Cancer Res 40, 308 (2021)**

**https://doi.org/10.1186/s13046-021-02103-5**

Following publication of the original article [1], the authors identified some minor errors in Fig. 9, specifically:

- In Fig. 9C, the image of SRC-3 in KD group; the incorrect image was used
- In Fig. 9D, the image of tumor in Sun group; the incorrect image was used

The corrected figures are given here.

**Fig. 9:**
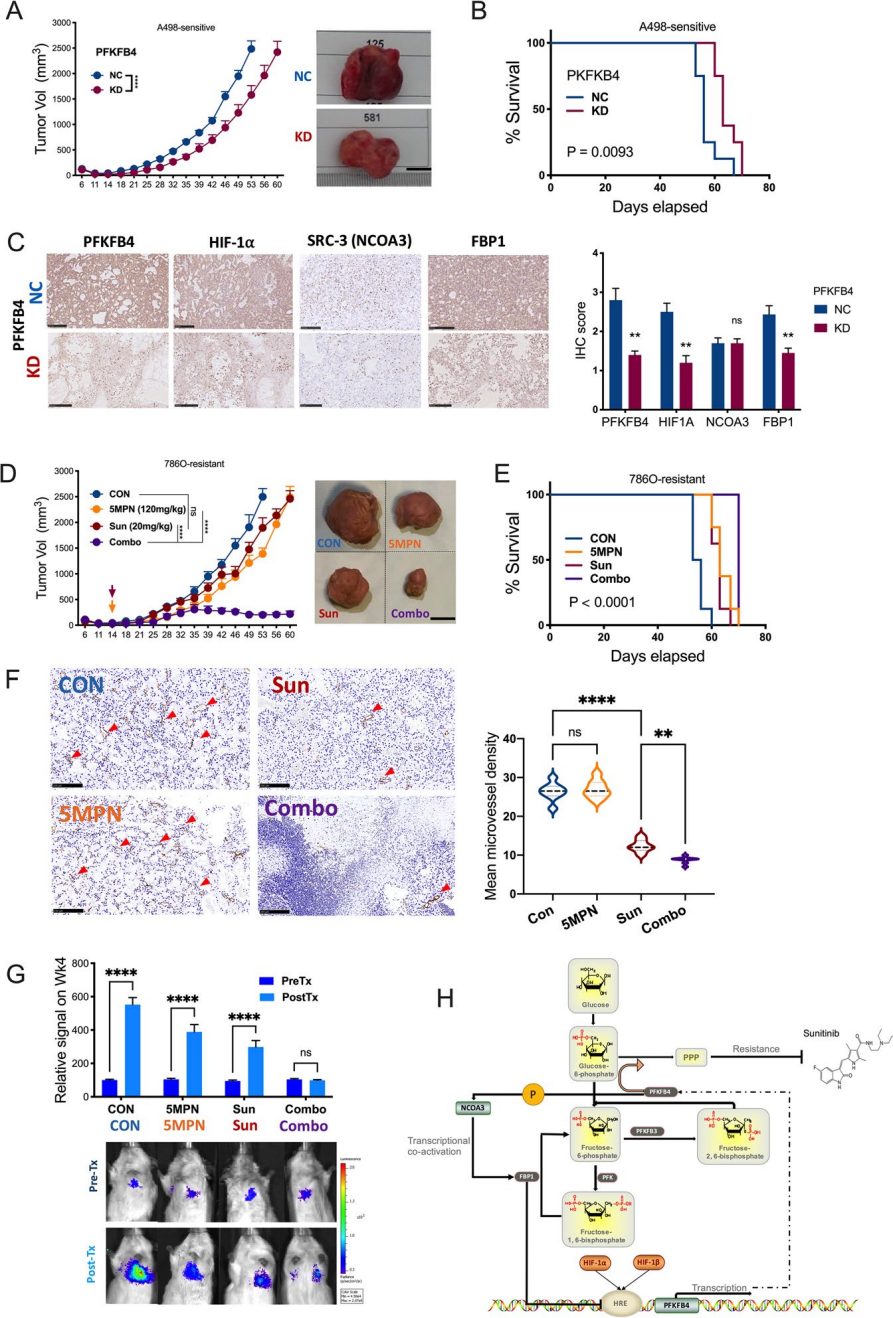
PFKFB4-knockdown overcomes Sunitinib resistance in ccRCC in vivo. **A** Xenograft murine models consisting of 8 male BALB/c nude mice per group with subcutaneous implanted Sun-sensitive A498 cells with or without PFKFB4-KD (shRNA#2) under right hind limb with tumor growth monitored over 60-day period and tumor size of < 2500 mm3 as endpoint, with representative tumor image at endpoint (bar = 1 cm) analyzed by two-way ANOVA and **B** Kaplan-Meier curves of survival of mice, analyzed by Log-rank test; **C** Representative immunohistochemical staining of factors in tumors from A), with intensity scored semi-quantitatively and statistically compared; **D** Xenograft murine models consisting of 8 male BALB/c nude mice per group with subcutaneous implanted Sun-resistant 786O cells fed with 20 mg/kg of Sun and/or 120 mg/kg of 5MPN orally by gavage; tumor growth monitored over 60-day period, analyzed by two-way ANOVA and **E** Kaplan-Meier curves of survival of mice, analyzed by Log-rank test; **F** Representative immunohistochemical staining CD31 targeting micro-vessels in tumors (red arrows) from **D**, with relative micro-vessel density (MVD) statistically compared; **G** Tail vein injection of Sun-resistant A498 cells in 9 mice per group with 5MPN, Sun or combo treatments (Tx); mice monitored for 4 weeks for photon detection, normalized to PreTx CON group; bar figures showing photon change before (PreTx) and after (PostTx) with representative luciferase image showing lung involvement at endpoint of each group **H** Schematic cartoon of regulatory axis proposed by the current study. (All in vitro assays performed in triplicates and at least 3 biological replicates; ns = not significant; *P < 0.05; **P < 0.01; ***P < 0.001; ****P < 0.0001)

In addition, the Acknowledgements section has been corrected; the updated text is as follows:

**Acknowledgements**

The authors thank Dr Guoqing Ji for critical technical support.

The corrections do not have any effect on the final conclusions of the paper. The original article has been corrected.

## References

[1] Feng C, Li Y, Li K (2021). PFKFB4 is overexpressed in clear-cell renal cell carcinoma promoting pentose phosphate pathway that mediates Sunitinib resistance. J Exp Clin Cancer Res.

